# PDZSeg: adapting the foundation model for dissection zone segmentation with visual prompts in robot-assisted endoscopic submucosal dissection

**DOI:** 10.1007/s11548-025-03437-7

**Published:** 2025-06-20

**Authors:** Mengya Xu, Wenjin Mo, Guankun Wang, Huxin Gao, An Wang, Ning Zhong, Zhen Li, Xiaoxiao Yang, Hongliang Ren

**Affiliations:** 1 Department of Electronic Engineering, CUHK, Hong Kong, China; 2 Shenzhen Research Institute, CUHK, Shenzhen, China; 3 Department of Computer Science and Engineering, Sun Yat-sen University, Guangzhou, China; 4 Department of Gastroenterology, Qilu Hospital of Shandong University, Jinan, China

**Keywords:** Dissection Zone Segmentation, Visual Prompts, Endoscopic Submucosal Dissection (ESD)

## Abstract

****Purpose**:**

The intricate nature of endoscopic surgical environments poses significant challenges for the task of dissection zone segmentation. Specifically, the boundaries between different tissue types lack clarity, which can result in significant segmentation errors, as the models may misidentify or overlook object edges altogether. Thus, the goal of this work is to achieve the precise dissection zone suggestion under these challenges during endoscopic submucosal dissection (ESD) procedures and enhance the overall safety of ESD.

****Methods**:**

We introduce a prompted-based dissection zone segmentation (PDZSeg) model, aimed at segmenting dissection zones and specifically designed to incorporate different visual prompts, such as scribbles and bounding boxes. Our approach overlays these visual cues directly onto the images, utilizing fine-tuning of the foundational model on a specialized dataset created to handle diverse visual prompt instructions. This shift toward more flexible input methods is intended to significantly improve both the performance of dissection zone segmentation and the overall user experience.

****Results**:**

We evaluate our approaches using the three experimental setups: in-domain evaluation, evaluation under variability in visual prompts availability, and robustness assessment. By validating our approaches on the ESD-DZSeg dataset, specifically focused on the dissection zone segmentation task of ESD, our experimental results show that our solution outperforms state-of-the-art segmentation methods for this task. To the best of our knowledge, this is the first study to incorporate visual prompt design in dissection zone segmentation.

****Conclusion**:**

We introduce the prompted-based dissection zone segmentation (PDZSeg) model, which is specifically designed for dissection zone segmentation and can effectively utilize various visual prompts, including scribbles and bounding boxes. This model improves segmentation performance and enhances user experience by integrating a specialized dataset with a novel visual referral method that optimizes the architecture and boosts the effectiveness of dissection zone suggestions. Furthermore, we present the ESD-DZSeg dataset for robot-assisted endoscopic submucosal dissection (ESD), which serves as a benchmark for assessing dissection zone suggestions and visual prompt interpretation, thus laying the groundwork for future research in this field. Our code is available at https://github.com/FrankMOWJ/PDZSeg.

## Introduction

Endoscopic Submucosal Dissection (ESD) is a surgical procedure employed in the treatment of early-stage gastrointestinal cancers. This procedure entails a complex series of dissection maneuvers that require significant skill to determine the dissection zone. In traditional ESD, a transparent cap is employed to retract lesions, which can often obscure the view of the submucosal layer and lead to an incomplete dissection zone. Conversely, our robot-assisted ESD [1] offers better visualization of the submucosal layer, resulting in a more completed dissection zone by utilizing robotic instruments that enable independent control over retraction and dissection. Achieving successful submucosal dissection requires the careful excision of the lesion or mucosal layer along with the complete submucosal layer while ensuring that both the underlying muscular layer and the mucosal surface remain unharmed. If the electric knife inadvertently contacts tissue outside the designated dissection zone (i.e., no-go zone), it can lead to damage to the muscle layer, increasing the risk of perforations. Such complications not only elevate the surgical risks but can also complicate the patient’s recovery. Therefore, it is imperative to maintain a precise dissection zone during endoscopic procedures. Effective guidance can help ensure that surgeons are adept at identifying and adhering to appropriate dissection boundaries and enhance the overall safety of endoscopic submucosal dissection (ESD).

However, the complexity of endoscopic surgical environments presents substantial challenges for the dissection zone segmentation task. Traditional segmentation algorithms often struggle to accurately identify relevant features within these intricate images because unrelated areas can easily mislead them. This tendency for confusion can lead to ambiguous segmentation outcomes, which fail to delineate the dissection area. Our work can also be applied to remote teaching and training in robotic surgery. A shortage of skilled expert surgeons, an uneven distribution of medical resources, the high costs associated with travel for remote training, and the growing demand for accessible and efficient surgical education collectively underscore the urgent need to develop intelligent, interactive, and precise remote assistance systems that align with the clinical demands of robotic surgery. Existing challenges in remote surgical live-streaming teaching include limited precision in remote guidance and inefficient use of expert time. Remote experts are often restricted to providing verbal instructions, which can be imprecise and open to misinterpretation. Without the ability to directly interact with the surgical environment or offer real time, hands-on guidance, the effectiveness of the training is significantly reduced. In addition, during surgery, it is common for surgeons to encounter situations where they must make quick decisions about dissection zones that can be safely operated. Without proper guidance, they may hesitate, leading to indecision that can prolong the procedure and increase the risk of adverse events. In a surgical setting, a seasoned physician with extensive experience and technical proficiency often provides guidance and visual prompts about the area that can be safely dissected to a less experienced colleague.

Drawing inspiration from the provided visual prompts, we propose training the models with their assistance. While existing deep learning-based segmentation models focus on whole image understanding, a prominent gap exists in achieving region-specific comprehension. Users can supply a visual prompt to indicate the region of interest (ROI), which refers to the specific area within an image that they wish to analyze or isolate. Many visual prompting systems have predominantly relied on regular shapes for visual prompts. However, we recognize the need for a model to interpret a broader spectrum of visual cues. Our research aims to enhance the interaction with models, making it more natural and intuitive. To address these challenges, we present an innovative segmentation model that adapts a foundation model to interpret various visual prompts. This design empowers users to intuitively plot images and engage with the model through natural cues, such as a “bounding box” or “scribble.” By directly overlaying visual prompts onto the RGB image, our approach eliminates the need for intricate prompt encodings while still achieving state-of-the-art performance for dissection zone segmentation.

We adopt the foundation models in this work because they have become a groundbreaking technique across multiple domains, utilizing extensive datasets to create generalized representations that can be fine-tuned for various downstream tasks. Their ability to recognize complex patterns and relationships in data makes them a strong foundation for numerous applications, providing an excellent starting point for training models tailored to specific needs. Given the advanced capabilities in extracting unified visual features, we have chosen DINOv2 [2] as the foundational backbone for developing the dissection zone segmentation model specifically designed for endoscopic surgery.

However, one of the challenges with foundation models is their tendency to experience a marked decline in predictive performance when applied to specialized domains. By injecting low-rank matrices into the model’s layers, Low-Rank Adaptation (LoRA) [3] allows for quick adjustments without extensive retraining, facilitating the use of foundation models in specialized segmentation tasks. This approach significantly reduces the computational and memory requirements for training, making the deployment of the model more feasible and efficient.

**Fig. 1:**
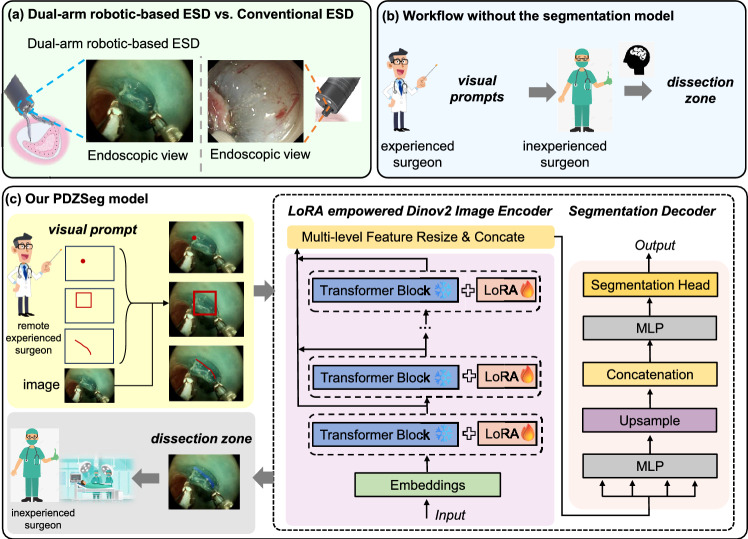
Overview. **a** Robotic ESD procedures were recorded from ex vivo porcine models utilizing our custom dual-arm robotic platform [1]. Robotic ESD provides improved visualization of the submucosal layer, leading to a more complete dissection zone compared to conventional ESD. **b** The detailed segmentation zone contour guidance is challenging for doctors to provide in real time. **c** We introduce a model, PDZSeg, specifically developed for segmenting dissection zones and capable of integrating different visual prompts provided by the experienced surgeon, such as scribbles and bounding boxes. Our approach incorporates these visual cues directly onto the images, leveraging fine-tuning of the foundational model. Our model then delivers precise dissection zone contours to the inexperienced surgeon

In summary, aligned with the clinical needs for intelligent, interactive, and precise remote assistance systems in robotic surgery, we aim to innovate the dissection zone suggestion scheme with the visual prompts solution. Our goal is to address critical technical challenges in identifying the dissection zone through remote teaching or expert guidance. Our method can streamline the training process by eliminating the need for complex prompt encodings and achieve more precise delineation of the dissection zone during endoscopic procedures, thereby significantly enhancing the overall effectiveness of the surgical intervention (Fig.  1). In this work, our contributions are summarized as follows:

- We present a prompted-based dissection zone segmentation (PDZSeg) model, designed for dissection zone segmentation and tailored to accommodate various visual prompts, including scribbles and bounding boxes. Our methodology involves overlaying these visual prompts directly onto the images, facilitated by fine-tuning the foundation model on our specialized dataset developed to manage a wide range of visual prompt instructions. This transition to more adaptable input methods seeks to enhance the dissection zone segmentation performance and user experience significantly.
- We propose a novel visual referral method for the dissection zone suggestion task that integrates visual prompts directly onto images, streamlining the model architecture while enhancing performance.
- We develop the ESD-DZSeg dataset for the dissection zone segmentation task for robot-assisted ESD from ex vivo porcine models using our custom dual-arm robotic system [1]. It can be regarded as a benchmark for evaluating dissection zone suggestion and assessing visual prompt interpretation, establishing a foundational framework for future research.

## Related work

**Segmentation models** Traditional segmentation models [4, 5] often struggle with complex images. Recent segmentation models have addressed the limitations of traditional methods by incorporating user inputs into the segmentation process. The integration of foundation models [6] into segmentation tasks has revolutionized the field. The Segment Anything Model (SAM) [6] requires a prompt embedding module to handle visual prompts. The Medical-SAM-Adapter [7] was developed to address SAM’s limitations in medical image segmentation, particularly its lack of domain-specific knowledge. Achieving precise segmentation, particularly in boundary areas, continues to pose challenges. To address this, guided contrastive boundary learning (GCBL) [8] is proposed to enhance the feature representation learning, leading to improved accuracy in boundary segmentation tasks.

**Fig. 2:**
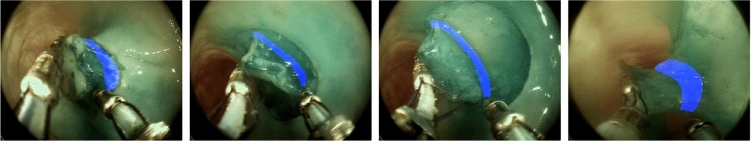
Our ESD-DZSeg dataset. The blue areas represent the ground truth of the dissection zone. The complexities of the endoscopic scene understanding task can be observed from the dataset. The features across various regions exhibit significant similarity, and the boundaries between these regions are often ambiguous

**Visual prompting systems** Traditional systems have predominantly relied on regular shapes for visual prompts. Many existing approaches [9] primarily utilize bounding box inputs for visual referrals. While this method is effective in structured environments, it falls short in more dynamic, user-driven interactions where visual prompts may not adhere to regular geometric forms. Recent advancements in interactive segmentation techniques demonstrate the potential for using points or scribbles as input [6].

## Methods

### Data collection and annotation

In this study, 19 robotic ESD procedures were collected from ex vivo porcine models using our custom dual-arm robotic system [1] in Qilu Hospital. The animal study was approved by the Institutional Ethics Committee (Approval No. DWLL-2021-021). Video recordings were captured at 30 FPS with $1920 \times 1080$ resolution using a flexible dual-channel endoscope. To isolate the surgical field of view by excluding the user interface (UI) elements, the endoscopic images were cropped to achieve a final resolution of $1310 \times 1010$. This research specifically targets the submucosal dissection task, which is characterized by extensive interactions with soft tissue and procedural intricacies. Video sequences pertinent to this task were selectively extracted and downsampled to 1 FPS to create the dissection dataset. We curated a total of 1, 849 images requiring dissection zone recommendations, sourced from 21 videos. These images were divided into two distinct subsets. Specifically, video 050744, video 030204, video 000032, and video 103115 comprised the validation set, which included 369 images. The remaining videos were allocated to the training set, totaling 1, 480 images. Expert endoscopists from Qilu Hospital were responsible for annotating the dissection zones for these images utilizing the annotation software LabelBox.^1^ Figure 2 illustrates our ESD-DZSeg (Dissection Zone Segmentation for ESD) dataset.

### Our dissection zone segmentation model

#### Visual prompts design

Our research aims to enhance the interaction with models, making it more natural and intuitive. Designing visual cues of varying intensities tailored to the needs of less experienced surgeons can significantly improve the confidence and safety of less experienced surgeons during surgery. Thus, we provide flexible visual prompt options. Users can provide inputs in various formats, such as: **points**: Drawing a single point to indicate a region of interest. **scribbles (long/short)**: Drawing freehand lines to specify the location of regions of interest (ROIs). **bounding boxes**: Encapsulating objects or areas within rectangular frames for easier identification. These natural visual prompts serve as cues for our dissection zone segmentation model, directing its attention to the specified ROIs, thus enhancing the efficiency and accuracy of the segmentation process. These visual cues can provided by the more experienced surgeon (see Fig. 1).

In the training phase, visual prompts are derived from professional surgeons’ annotations: scribble prompts from optimal dissection trajectories, bounding boxes from the minimum rectangles around safe areas, and point prompts from random points within safe areas. In the inference phase, prompts are provided by doctors in real time. The visual prompts are superimposed onto the RGB images by mapping specific regions of the original images to predefined color codes representing the prompts. These augmented images are then directly input into the model for subsequent processing.

#### Dissection zone segmentation model

Essentially, our segmentation model suggests the dissection zone for these inexperienced doctors, emphasizing the detailed delineation of critical areas that may be challenging for experienced professionals to guide in real time. The proposed Prompted-based Dissection Zone Segmentation (PDZSeg) model comprises three primary components: an image encoder, a LoRA efficient training module, and a segmentation decoder. The module processes the RGB raw images attached with the visual prompt and generates the dissection zone masks.

**Image encoder.** DINOv2 [2] is a state-of-the-art self-supervised vision foundation model, designed to learn powerful image representations from large-scale unlabeled datasets. We adopt the pre-trained DINOv2 as our image encoder, which can generate multi-level, rich feature representations that enhance the subsequent segmentation task. To be specific, an input image $x \in \mathbb {R} ^{H\times W \times C}$ first be embedded into non-overlapping patches $z_k^0 \in \mathbb R ^ {N \times D}$, $1 \le k \le N$, where $N = \frac{HW}{p^2}$, *p* is the size of patch and *D* is the dimension of each patch, attached with an additional class token $z_0^0 \in \mathbb R ^{1 \times D}$, following the practical in Vision Transformer(ViT) [10]. The combined tokens $z^0 = [z^0_0, z^0_1, ..., z^0_k]$ are then passed through *M* transformer blocks to extract intermediate feature representations, denoted as $z^m$ for $1 \le m \le M$. At each selected stage, the intermediate feature representations, consisting of both the class token and image tokens, are concatenated along the channel dimension and upsampled by a factor of 4. The features from all the selected stages are then combined to form the final image representation for the decoder.

**LoRA efficient training module.** For efficient training, we only add the LoRA layer to the $W_q$ and $W_v$ matrices in each of the attention blocks. Specifically, let $x^{t}_{i}$ represent a token embedding at the *t*th transformer block, and $W^{t}_k$, $W^{t}_q$, and $W^{t}_v$ be the key, query, and value projection matrices of the transformer block, respectively. Let $A_q$, $B_q$, $A_v$, and $B_v$ denote the LoRA low-rank matrices corresponding to $W^{t}_q$ and $W^{t}_v$. The attention scores can then be calculated as: $\text {Att}(Q, K, V) = \text {Softmax} \left( \frac{QK^T}{\sqrt{D}} \right)$, $Q = W_q x + B_q A_q x$, $K = W_k x$, $V = W_v x + B_v A_v x$, and *D* is the numbers of output tokens.

**Segmentation decoder.** A lightweight All-MLP network is utilized as the segmentation decoder. The decoder utilizes three MLP layers to process and transform features extracted from the image encoder. The first MLP layer aligns *n* encode features to the same dimension $C$, followed by upsampling by a factor of 4 and concatenation. The second MLP layer then transforms the concatenated features with a dimension of $n \times C$ to $C$. Finally, the third MLP layer maps the fused *C*-dimensional features to the $N_{cls}$-dimensional segmentation mask, where $N_{cls}$ is the number of classes.

## Experiments

### Implementation details

We implement our models using the PyTorch framework and conduct all experiments on a single NVIDIA RTX 4090 GPU. To meet the requirements of the foundation model DINOv2, which processes inputs with sizes that are multiples of 14, the images and their corresponding segmentation masks are resized to 532 $\times$ 532 using the *torchvision.transforms.Resize* function with bilinear interpolation. Vit-Base with 12 transformer blocks and feature dimension of 784 is adopted as the backbone of DINOv2 [2]. Intermediate outputs from $m = {3, 6, 9, 12}$ transformer blocks are extracted as image representations. A pre-trained checkpoint is loaded for the DINOv2 encoder, while the segmentation decoder is randomly initialized. The encoder is fine-tuned using LoRA [3] with rank of 4, and the decoder is trained from scratch with a learning rate of 0.001. The batch size is set to 8. We train the models for 100 epochs using the Adam optimizer with cross-entropy (CE) loss and the Cosine Annealing scheduler.

### Results and analysis

**Table 1:** The dissection zone segmentation performance of different models, with and without various visual prompts. The PDZSeg results include improvement values in parentheses: the first denotes improvement over the worst baseline, and the second over the best. Note that positive improvement values in ASSD and HD indicate better metric performance

Visual Prompt	Model	Dissection zone		No-go zone		Segmentation metrics		Boundary metrics	
		IoU$\uparrow$	Dice$\uparrow$	IoU$\uparrow$	Dice$\uparrow$	Mean IoU$\uparrow$	Mean Dice$\uparrow$	ASSD$\downarrow$	HD$\downarrow$
Without visual prompt	SETR	43.91	61.02	97.93	98.95	70.92	79.99	38.51	9.31
	STDC	41.92	59.07	97.79	98.88	69.85	78.97	45.15	9.72
	Point-Rend	44.71	61.79	97.65	98.81	71.18	80.30	38.85	9.46
	Fast-SCNN	41.86	59.01	97.77	98.87	69.81	78.94	39.50	9.47
	DeepLabv3	45.95	62.97	97.90	98.94	71.93	80.95	38.47	9.17
	Our PDZSeg	**47.55 (5.69/1.60)**	**63.01 (4.0/0.04)**	**98.05 (4.0/0.12)**	**99.04 (0.23/0.09)**	**72.80 (2.99/0.87)**	**81.02 (2.08/0.07)**	**31.37 (13.78/7.1)**	**8.78 (0.94/0.39)**
With bbox visual prompt	SETR	64.48	78.40	98.75	99.37	81.61	88.89	15.67	7.52
	STDC	69.41	81.94	98.90	99.45	84.15	90.69	13.39	7.21
	Point-Rend	67.87	80.86	98.80	99.40	83.33	90.13	14.29	7.35
	Fast-SCNN	66.36	79.78	98.73	99.36	82.55	89.57	14.93	7.36
	DeepLabv3	69.24	81.83	98.90	99.45	84.07	90.64	13.30	**7**.**05**
	SAM-adapter	64.41	78.18	98.69	99.34	81.54	88.75	15.03	7.76
	MedSAM	48.62	60.81	98.28	99.13	73.44	79.96	23.67	8.68
	SAM_Med2D	57.53	72.41	98.50	99.24	78.01	85.82	18.81	8.08
	Our PDZSeg	**69.85 (21.23/0.44)**	**82.00 (21.19/0.06)**	**98.97 (0.69/0.07)**	**99.48 (0.35/0.03)**	**84.41 (10.97/0.26)**	**90.73 (10.77/0.04)**	**12.56 (11.11/0.74)**	**7.05 (1.63/0.00)**
With short scribble visual prompt	SETR	62.38	76.83	98.66	99.33	80.52	88.08	17.81	7.82
	STDC	62.65	77.04	98.58	99.28	80.61	88.16	18.70	8.29
	Point-Rend	64.42	78.36	98.74	99.37	81.58	88.86	16.84	7.98
	Fast-SCNN	61.43	76.11	98.60	99.30	80.02	87.70	18.18	8.04
	DeepLabv3	64.15	78.16	98.73	99.36	81.44	88.76	16.96	7.95
	Our PDZSeg	**65.45 (4.02/1.03)**	**78.41 (2.3/0.05)**	**98.83 (0.25/0.09)**	**99.41 (0.13/0.04)**	**82.14 (2.12/0.56)**	**88.91 (1.21/0.05)**	**15.26 (3.44/1.58)**	**7.71 (0.58/0.11)**
With point visual prompt	SETR	50.53	67.14	98.18	99.08	74.36	83.11	28.70	8.82
	STDC	51.64	68.11	98.21	99.10	74.93	83.61	29.07	9.02
	Point-Rend	54.49	70.54	98.25	99.12	76.37	84.83	27.76	8.57
	Fast-SCNN	51.51	68.00	98.25	99.12	74.88	83.56	28.08	9.02
	DeepLabv3	55.57	71.39	98.37	99.18	76.97	85.28	25.69	8.58
	SAM-adapter	39.31	55.44	97.51	98.73	68.41	76.96	41.97	9.72
	SAM_Med2D	44.32	60.32	98.00	98.98	71.16	79.65	33.21	9.29
	Our PDZSeg	**56.37 (17.06/0.80)**	**71.43 (15.99/0.04)**	**98.46 (0.95/0.09)**	**99.22 (0.49/0.04)**	**77.41 (9.00/0.44)**	**85.32 (8.36/0.04)**	**22.41 (19.56/3.28)**	**8.29 (1.43/0.28)**
With long scribble visual prompt	SETR	70.85	82.94	98.97	99.48	84.91	91.21	13.13	6.75
	STDC	70.19	82.49	98.94	99.47	84.56	90.98	13.38	6.87
	Point-Rend	**74**.**73**	**85**.**54**	**99**.**17**	**99**.**58**	**86**.**95**	**92**.**56**	**10**.**52**	**6**.**46**
	Fast-SCNN	71.87	83.63	99.04	99.52	85.46	91.58	12.14	6.59
	DeepLabv3	72.30	83.93	99.08	99.54	85.69	91.73	11.64	6.55
	Our PDZSeg	74.06 ($-$0.67/3.87)	84.3 ($-$1.24/1.81)	99.15 ($-$0.02/0.21)	99.56 ($-$0.02/0.09)	86.60 ($-$0.35/2.04)	91.93 ($-$0.63/0.95)	10.72 ($-$0.2/2.66)	6.53 ($-$0.07/0.34)

**Model evaluation with and without visual prompts** To evaluate the performance of our proposed method, several state-of-the-art (SOTA) models, such as SETR [11], STDC [12], Point-Rend [4], Fast-SCNN [13], DeepLabv3 [5], Medical-SAM-Adapter [7], MedSAM [14], and SAM-Med2D [15], are used as our baselines. The area outside the dissection zone is termed the “No-go zone”.

IoU, Dice, Average Symmetric Surface Distance (ASSD) [16], and the Hausdorff Distance (HD) [16] are used as our evaluation metrics.

**Fig. 3:**
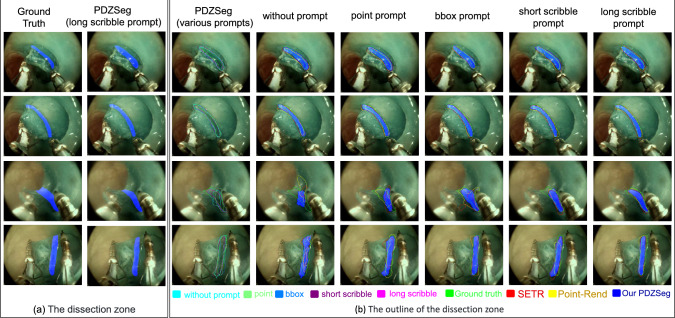
Results visualization. The first two columns display the ground truth and the segmentation masks predicted by our model. To enhance the comparison, we have extracted the outlines of the segmentation masks, which more clearly highlight the boundaries of the dissection zones. The differently colored contours represent the results obtained from corresponding methods

Table 1 summarizes the segmentation performance of various models, highlighting the following key findings: (a) When we revise our model (PDZSeg) to a version that does not utilize visual cues (DZSeg), it still achieves better segmentation results than most other models, outperforming the baseline models for at least $1.6 \%$ in IoU of the dissection zone. (b) These baseline models do not inherently incorporate visual prompt designs; however, their performance has significantly improved when combined with our visual prompt approach. For DeepLabv3, the Mean IoU improves by $5.04\%$, $9.51\%$, $12.14\%$, and $13.76\%$ when integrated with point, short-scribble, bounding-box, and long-scribble prompts, respectively. (c) The results obtained with visual prompts are substantially better than those without. This indicates that segmentation performance improves notably when visual prompts direct the model toward specific regions of interest. (d) Among the four forms of visual prompts we designed, the model employing long-scribble prompts exhibits the highest performance, followed by the model using bounding-box prompts and short-scribble prompts. The model utilizing point prompts produces the least favorable results. For our PDZSeg, the Mean IoU is $86.60\%$, $84.41\%$, $82.12\%$, and $77.41\%$ when incorporated with long-scribble, bounding-box, short-scribble, and point visual prompts, respectively (Fig. 3).

**Table 2:** Our model evaluation under a diverse dataset of prompted and non-prompted scenarios in ratios of 6:4, 5:5, and 4:6

Visual prompt	with : without	Dissection zone		No-go zone		Mean IoU	Mean Dice
		IoU	Dice	IoU	Dice		
long-scribble visual prompt	6–4	73.41	83.98	99.12	99.56	86.27	91.76
	5–5	73.46	83.87	99.13	99.56	86.31	91.72
	4–6	71.81	83.08	99.07	99.53	85.45	91.3
short-scribble visual prompt	6–4	58.04	71.49	98.52	99.25	78.28	85.37
	5–5	54.72	68.7	98.43	99.2	76.58	83.97
	4–6	52.51	66.91	98.32	99.15	75.41	83.03
bounding-box visual prompt	6–4	58.96	72.04	98.89	99.43	78.92	85.73
	5–5	56.85	70.87	98.45	99.21	77.65	85.04
	4–6	55.2	68.93	98.39	99.18	76.79	84.06

**Model evaluation under variability in visual prompt availability:** We also integrate a diverse dataset that includes both prompted and non-prompted scenarios to represent the complexities inherent in real-world clinical environments more accurately. The integration ratios of prompt and non-prompt data are 6:4, 5:5, and 4:6, respectively. In clinical practice, even seasoned physicians may encounter situations where they cannot provide comprehensive prompts for every case, leading to variability in the information available for decision-making. The assessment outcomes are displayed in Table 2. The variation in model performance, as the ratio of with-prompts to without-prompts shifts from 6 : 4 to 4 : 6 remains within a range of $2.81\%$ in Mean IoU and $2.34\%$ in Mean Dice, which is considered acceptable. Moreover, we also integrate a blend of data that includes a variety of prompt types alongside non-prompted scenarios, reflecting the range of responses that physicians might offer in different contexts. Data prompts with long scribbles, short scribbles, bounding boxes, and data with no prompts account for $25\%$. This variability in prompt types is crucial, as it acknowledges that clinicians may have differing levels of insight or detail depending on the specific circumstances of each case. The evaluation findings are illustrated in Table 3. Compared to the results in Table 1, the IoU of the dissection zone decreases by only $3.38\%$, $5.27\%$, $4.9\%$, and $3.32\%$ when using long scribbles, short scribbles, bounding boxes, and no prompts, respectively.

**Table 3:** Our model evaluation under a dataset that includes various prompt types and non-prompted scenarios, with each type comprising 25% of the data

Visual prompt in train set	Visual prompt in val set	Dissection zone		No-go zone		Mean IoU	Mean Dice
		IoU	Dice	IoU	Dice		
Mix visual prompt	Long scribble	70.68	81.86	99.00	99.49	84.84	90.67
	Short scribble	60.18	74.84	98.65	99.31	79.41	87.08
	Bounding box	64.92	78.27	98.78	99.38	81.85	88.82
	Without prompt	44.23	59.13	97.96	98.96	71.09	79.05

**Fig. 4:**
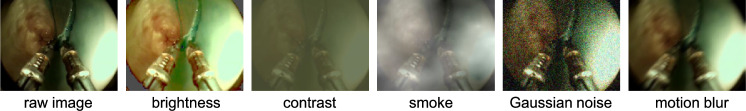
Corrupted images at a severity level of 3

**Table 4:** Performance of dissection zone segmentation under five different image corruption techniques

Visual prompt	Model	Gaussian noise		Motion blur		Smoke		Contrast		Brightness	
		IoU	Dice	IoU	Dice	IoU	Dice	IoU	Dice	IoU	Dice
without visual	Point-Rend	1.94	3.81	22.28	36.44	5.58	10.56	0.51	1.02	37.08	54.10
	Our PDZSeg	21.45	31.62	25.15	36.01	24.22	36.07	37.30	50.44	46.21	60.61
prompt point	Point-Rend	13.76	24.19	37.60	54.65	27.19	42.75	23.44	37.97	49.41	66.14
	Our PDZSeg	24.15	36.41	35.34	50.11	28.58	44.57	44.68	61.24	54.21	68.81
bounding box	Point-Rend	16.21	27.90	55.65	71.51	45.00	62.07	43.76	60.88	56.56	74.66
	Our PDZSeg	43.35	56.40	57.14	72.09	60.06	75.01	65.54	78.62	68.70	81.07
short scribble	Point-Rend	4.98	9.49	45.27	62.32	42.58	59.73	44.03	61.14	56.13	71.90
	Our PDZSeg	45.34	57.43	58.81	72.73	59.67	73.98	61.74	75.85	64.66	77.53
long scribble	Point-Rend	2.89	5.61	65.93	79.47	62.28	76.76	64.90	78.72	70.66	82.81
	Our PDZSeg	64.91	77.11	70.09	80.80	70.73	81.21	72.01	82.57	73.52	83.41

**Robustness evaluation** The overall purpose of robustness evaluation is to assess how well the model maintains reliable performance under challenging conditions, such as image corruption, ensuring its practical applicability and safety in real-world surgical scenarios. As illustrated in Fig. 4, we apply five types of image corruption techniques to our validation set, each at a severity level of 3. These techniques include Gaussian noise, motion blur, smoke, brightness, and contrast adjustments [17]. The evaluation results are presented in Table 4. Compared with Point-Rend, when facing Gaussian noise, our model outperforms it by $62.02 \%$, $27.14 \%$, $40.36 \%$, $10.39 \%$, and $19.51 \%$ when integrating with long-scribble, bounding-box, short-scribble, point, and no prompts, demonstrating the robustness of our method.

## Conclusion

We introduce the prompted-based dissection zone segmentation (PDZSeg) model, which is specifically designed for dissection zone segmentation and accommodates various visual prompts such as scribbles and bounding boxes. By overlaying these visual prompts onto images and fine-tuning the foundation model on a specialized dataset, we aim to significantly enhance both segmentation performance and user experience. Our novel visual referral method further streamlines the model architecture while improving effectiveness in dissection zone suggestion tasks. Additionally, we have developed the ESD-DZSeg dataset for robot-assisted endoscopic submucosal dissection (ESD) using ex vivo porcine models with our custom dual-arm robotic system. This dataset serves as a benchmark for evaluating dissection zone suggestions and visual prompt interpretation, establishing a foundational framework for future research in this domain.

## Data Availability

https://github.com/FrankMOWJ/PDZSeg.

## Footnotes

1 https://labelbox.com/

## Declarations

### Code Availability

https://github.com/FrankMOWJ/PDZSeg.
